# Identification of Immune Infiltration in Odontogenic Keratocyst by Integrated Bioinformatics Analysis

**DOI:** 10.1186/s12903-023-03175-9

**Published:** 2023-07-06

**Authors:** Nian-Nian Zhong, Su-Ran Li, Qi-Wen Man, Bing Liu

**Affiliations:** 1 State Key Laboratory of Oral & Maxillofacial Reconstruction and Regeneration, Key Laboratory of Oral Biomedicine Ministry of Education, Hubei Key Laboratory of Stomatology, School & Hospital of Stomatology, Wuhan University, Wuhan, China; 2 Department of Oral & Maxillofacial - Head Neck Oncology, School & Hospital of Stomatology, Wuhan University, Wuhan, China

**Keywords:** Odontogenic keratocyst, Immune cell infiltration, Bioinformatics, Hub gene

## Abstract

**Background:**

Odontogenic keratocyst (OKC) is a relatively common odontogenic lesion characterized by local invasion in the maxillary and mandibular bones. In the pathological tissue slices of OKC, immune cell infiltrations are frequently observed. However, the immune cell profile and the molecular mechanism for immune cell infiltration of OKC are still unclear. We aimed to explore the immune cell profile of OKC and to explore the potential pathogenesis for immune cell infiltration in OKC.

**Methods:**

The microarray dataset GSE38494 including OKC and oral mucosa (OM) samples were obtained from the Gene Expression Omnibus (GEO) database. The differentially expressed genes (DEGs) in OKC were analyzed by R software. The hub genes of OKC were performed by protein-protein interaction (PPI) network. The differential immune cell infiltration and the potential relationship between immune cell infiltration and the hub genes were performed by single-sample gene set enrichment analysis (ssGSEA). The expression of COL1A1 and COL1A3 were confirmed by immunofluorescence and immunohistochemistry in 17 OKC and 8 OM samples.

**Results:**

We detected a total of 402 differentially expressed genes (DEGs), of which 247 were upregulated and 155 were downregulated. DEGs were mainly involved in collagen-containing extracellular matrix pathways, external encapsulating structure organization, and extracellular structure organization. We identified ten hub genes, namely FN1, COL1A1, COL3A1, COL1A2, BGN, POSTN, SPARC, FBN1, COL5A1, and COL5A2. A significant difference was observed in the abundances of eight types of infiltrating immune cells between the OM and OKC groups. Both COL1A1 and COL3A1 exhibited a significant positive correlation with natural killer T cells and memory B cells. Simultaneously, they demonstrated a significant negative correlation with CD56dim natural killer cells, neutrophils, immature dendritic cells, and activated dendritic cells. Immunohistochemistry analysis showed that COL1A1 (*P* = 0.0131) and COL1A3 (*P* < 0.001) were significantly elevated in OKC compared with OM.

**Conclusions:**

Our findings provide insights into the pathogenesis of OKC and illuminate the immune microenvironment within these lesions. The key genes, including COL1A1 and COL1A3, may significantly impact the biological processes associated with OKC.

**Supplementary Information:**

The online version contains supplementary material available at 10.1186/s12903-023-03175-9.

Tel./Fax: +86 87,686,215.

## Introduction

Odontogenic keratocyst (OKC) is a relatively common and locally aggressive cystic lesion accounting for approximately 8–11% of all jaw cyst lesions [1, 2]. High recurrence rates dependent of various modalities were reported [3]. Our previous study revealed that high numbers of T cells, macrophages, neutrophils and B cells were found in patients with OKC, as well as low numbers of dendritic cells (DCs), NK cells and innate lymphoid cells [4]. Especially in fibroblasts of OKC, various kinds of chemokines, including CXCL1, CXCL8, CXCL6, CXCL13 and CXCL2, were highly enriched and activated, which are vital for immune cell infiltration and angiogenesis [4]. In addition to cells, non-cellular components, namely extracellular matrix (ECM), can also influence cell fate and function via cell-matrix interactions [5]. However, the accurate mechanism of immune cell infiltration in OKC has not been fully explored.

The ECM is a complex network of various macromolecules surrounding the cells within the body [6]. Collagens are the main constituents of the ECM comprising around 30% of the whole protein mass [7]. A study has indicated that all hallmarks of cancer are potentially influenced by tumor-associated changes to the ECM [8]. In cancer, remodeling of the ECM induces various biophysical and biochemical changes affecting cell signaling, ECM stiffness, cell migration and tumor progression [9]. Collagen, especially collagen type I, was reported to directly or indirectly regulate the activity of a few types of immune cells, such as T cells and macrophages [6]. However, the mechanism by which collagen components influence immune cell infiltration in OKC tissues has not been fully elucidated.

The main objective of this study was to investigate the molecular mechanisms implicated in OKC development, emphasizing the role of immune cells in its pathogenesis. To this end, we conducted a comprehensive bioinformatics analysis of differentially expressed genes (DEGs) sourced from the GSE38494 dataset. The purpose of this detailed analysis was to pinpoint crucial hub genes and provide an exhaustive understanding of the immune cell infiltration patterns characteristic of OKC.

## Materials and methods

### Study Design

**Fig. 1:**
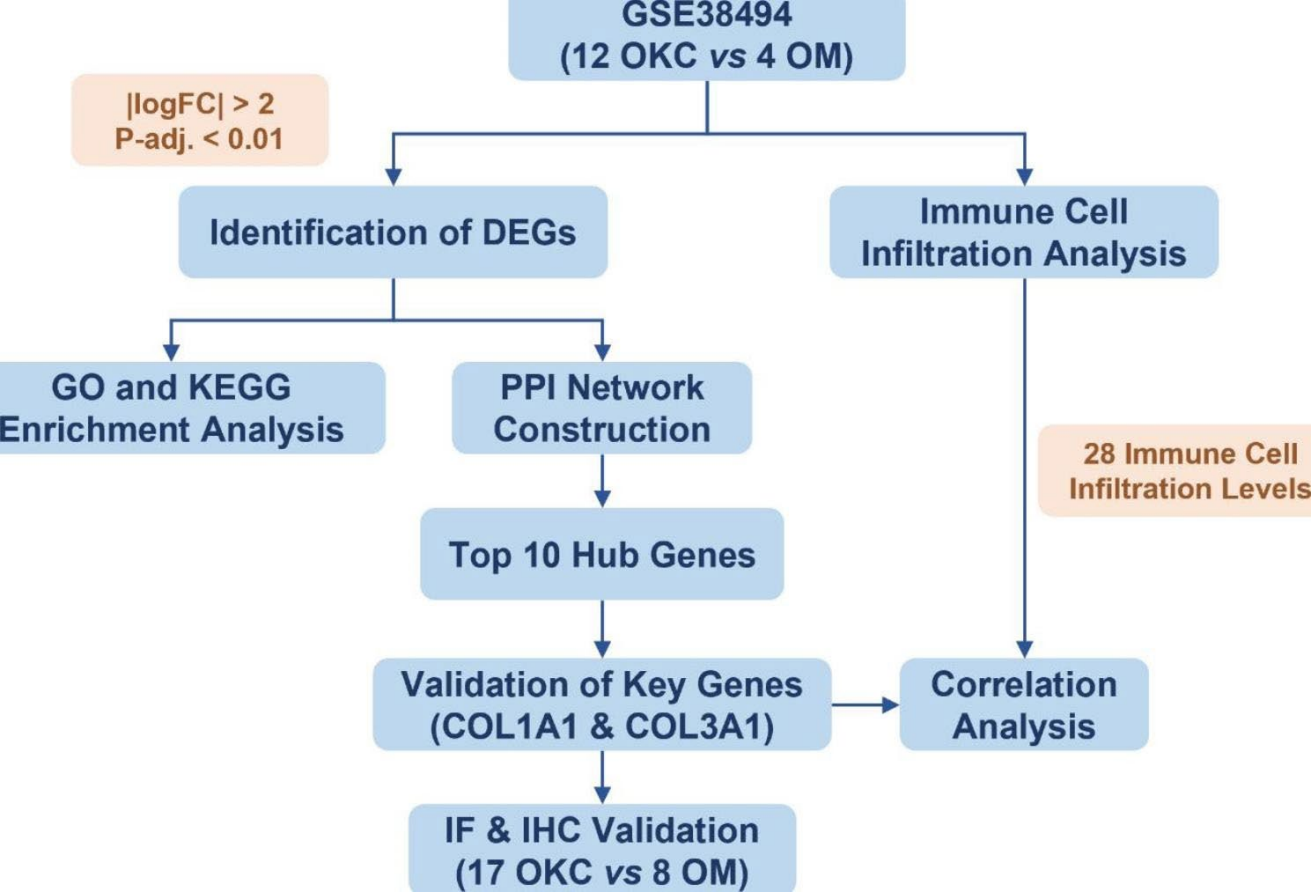
The flowchart of present study

As shown in Fig. 1, we used the array dataset GSE38494 from the Gene Expression Omnibus (GEO) to study DEGs. A series of bioinformatics analyses, including DEG screening, functional enrichment analysis, protein-protein interaction (PPI) analysis for the identification of hub genes, single sample gene set enrichment analysis (ssGSEA) for further analysis of immune cell infiltration, and correlation analysis between the expression levels of hub genes and the infiltration abundance of immune cells, were performed. Lastly, the expression of hub genes and abundance of immune cells were verified by immunohistochemistry and immunofluorescence in tissue microarray.

### Raw Data Collection

GSE38494 [including 12 OKCs (not associated with nevoid basal cell carcinoma syndrome) and 4 oral mucosa (OM) samples] annotated by the GPL570 platform, were downloaded from the GEO database.

### Test for correlation and variation of samples

R software (version 4.1.3) was used for the informatics analysis in this study. To explore the correlation of the samples in GSE38494, Pearson’s correlation test was performed using the “cor.test” function, and a correlation heatmap was drawn to visualize correlations between samples using the “heatmap” package of R. Principal component analysis (PCA) was used to visualize the variation and clustering of samples using the “FactoMinerR” package of R.

### Identification of DEGs

The “limma” package was used to normalize and screen DEGs between OM samples and OKC samples. DEGs with an adjusted *P* < 0.01 and a |log fold change (FC)| > 2 were considered significant. A heatmap and a volcano plot were drawn using R to visualize the DEGs.

### Enrichment Analysis

The “clusterProfiler” package was used for Gene Ontology (GO) [10] term and Kyoto Encyclopedia of Genes and Genomes (KEGG, www.kegg.jp/kegg/kegg1.html) [11, 12] pathway enrichment analyses employing DEGs and the *p*-value cutoff was set to 0.05.

### Construction of the PPI Network and Identification of hub genes

The online Search Tool for the Retrieval of Interacting Genes (STRING, https://www.string-db.org/) database was used to construct the DEG PPI network, with a PPI score threshold (medium confidence) ≥ 0.4. The cytoHubba plugin in Cytoscape (version 3.8.2, https://cytoscape.org/) was used to identify hub genes using the degree method (top 10 genes).

### Single sample gene set Enrichment Analysis of infiltrating Immune cells

The estimated proportion of 28 immune cell types in all samples (normal: 4, OKC: 12) of the GSE38494 dataset were calculated using R package “GSVA”. The estimated proportions of each immune cell type were visualized using the “ggplot2”. The Pearson’s correlation between the proportions of various types of immune cells in all the samples was calculated using the “cor.test” function of R, as well as the Pearson’s correlation between the immune cell abundance of the samples and top 10 hub genes.

### Immunofluorescence staining

Immunofluorescence (IF) staining were performed as previously described [13]. In brief, the Sect. (4 μm) were dewaxed, rehydrated, antigenretrieved, blocked, and incubated with the primary anti-COL1A1 antibody (1:200; Proteintech, 67288-1-lg) or anti-COL3A1 antibody (1:200; GeneTex, GTX102997) at 4℃ overnight, respectively. The secondary antibody (goat anti-rabbit-IgG-Alexa Fluor 488, Abbkine, A23220) were incubated at 37 °C for 20 min, respectively. The slides were then sealed by Antifade Mounting Medium with DAPI (Beyotime, P0131).

### Immunohistochemistry staining

Immunohistochemistry (IHC) staining were performed as previously described [13]. Eight OM and seventeen OKC samples were used. In brief, the Sect. (4 μm) were dewaxed, rehydrated, antigenretrieved, blocked, and incubated with the primary anti-COL1A1 antibody (1:2500; Proteintech, 67288-1-lg) or anti-COL3A1 antibody (1:200; GeneTex, GTX102997) at 4℃ overnight. Subsequently, the secondary antibodies incubation and DAB staining were performed. The sections were observed and scanned with the Aperio ScanScope CS scanner and analyzed by the Aperio Quantification software. H-scores (histo-scores) of each slide were calculated as previously reported [14].

### Statistical analysis

R software (version 4.1.3) was used to perform bioinformatics analyses. GraphPad Prism software (version 9.3.1) was used to analyze experimental data. Unpaired Student’s t-test was used to compare the two sets of data. *P* < 0.05 was considered statistically significant if not particularly indicated.

## Results

### Dataset validation

**Fig. 2:**
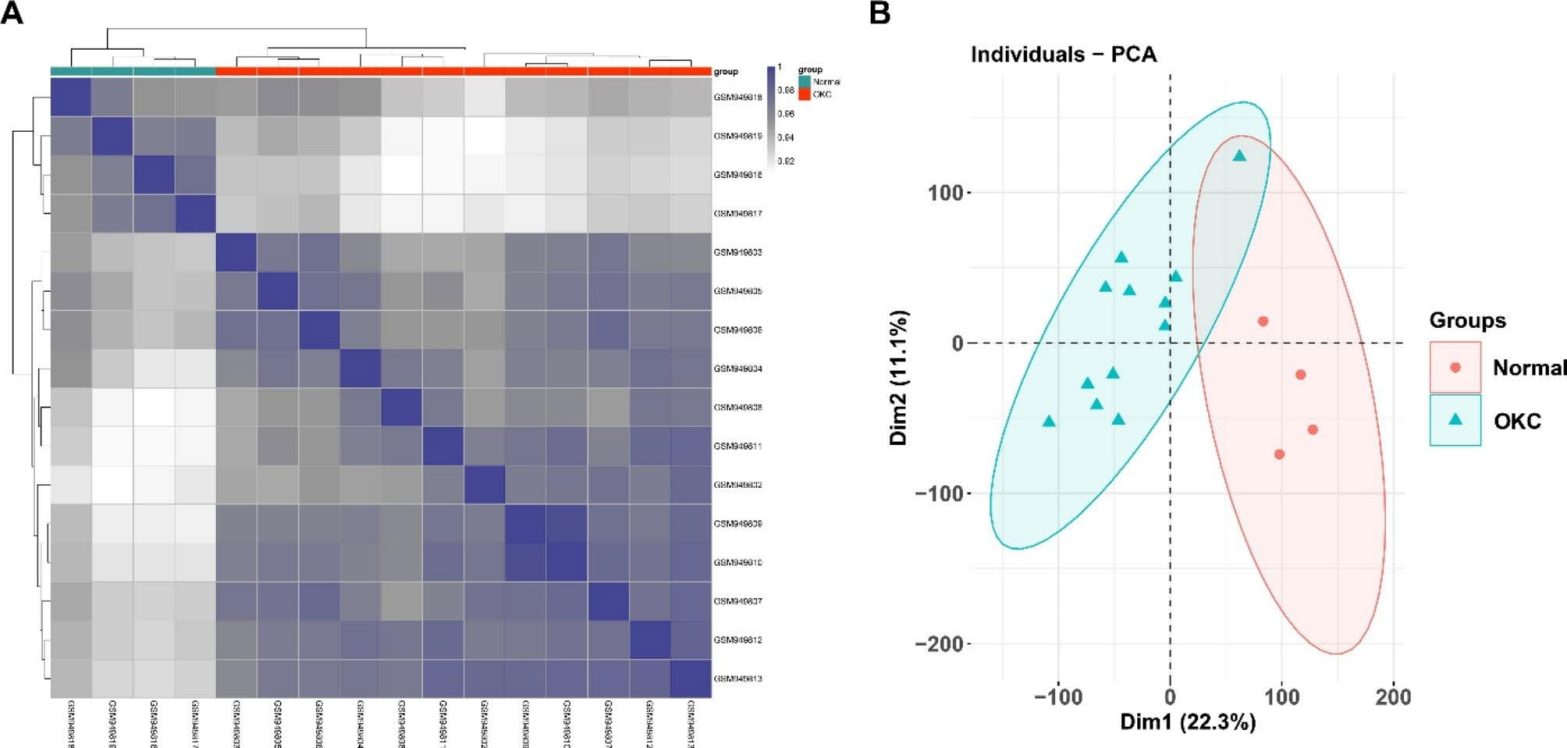
Validation of the dataset GSE38494 by Pearson’s correlation analysis (**A**) and PCA (**B**)

Pearson’s correlation test and PCA were used to validate the dataset. The correlation heatmap of the GSE38494 dataset showed that there were strong correlations among samples within the OM group as well as within the OKC group (Fig. 2A). PCA of GSE38494 showed that the 16 samples in the two groups could be distinguished and samples within a same group showed a closer distance. PC1 accounted for 22.3% of the variance and PC2 accounted for 11.1% of the variance (Fig. 2B).

### Differential Analysis

**Fig. 3:**
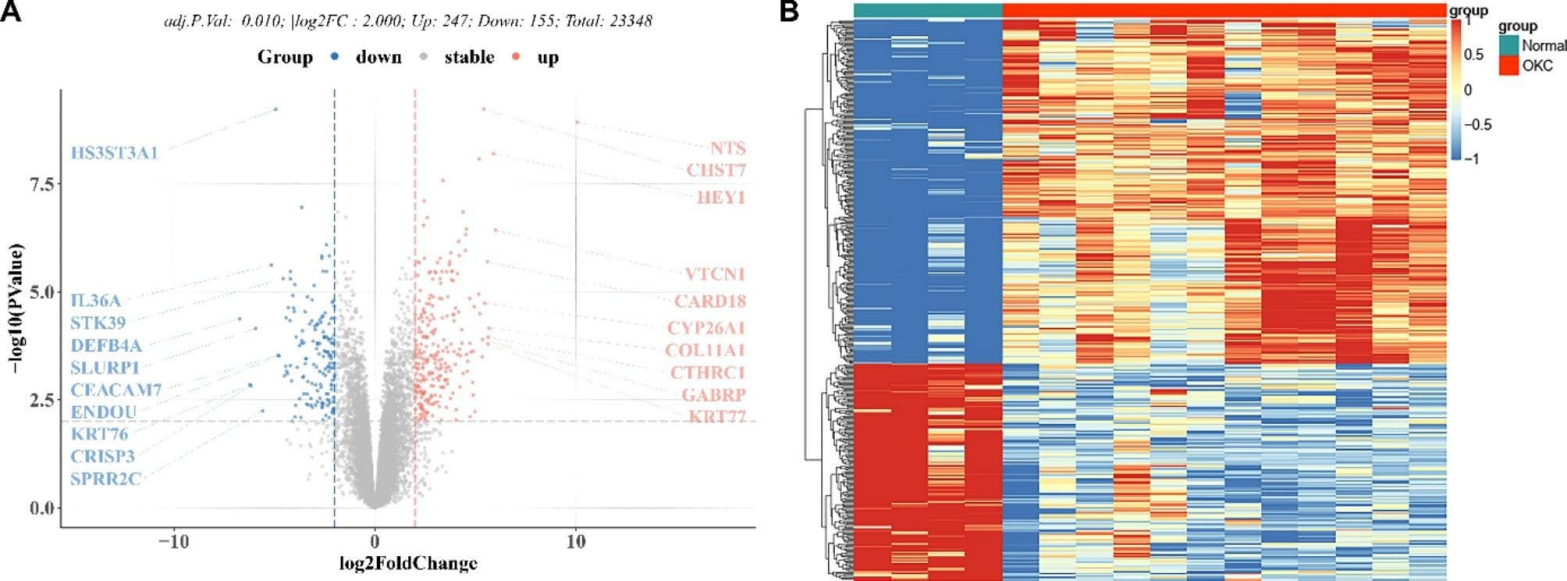
Identification of DEGs between oral mucosa (OM) samples and odontogenic keratocyst (OKC) samples. Volcano plot (**A**) and heatmap (**B**) showed the differentially expressed genes of GSE38494.

The R package “limma” was used for differential analysis of the screened expression profiles. A total of 402 DEGs were identified (**Supplementary Table 1**), among which 247 genes were up-regulated and 155 genes were down-regulated in OKC group when compared to the OM group, as presented in volcano plot (Fig. 3A) with top 10 genes indicated and heatmap (Fig. 3B).

### Function Enrichment Analysis

**Fig. 4:**
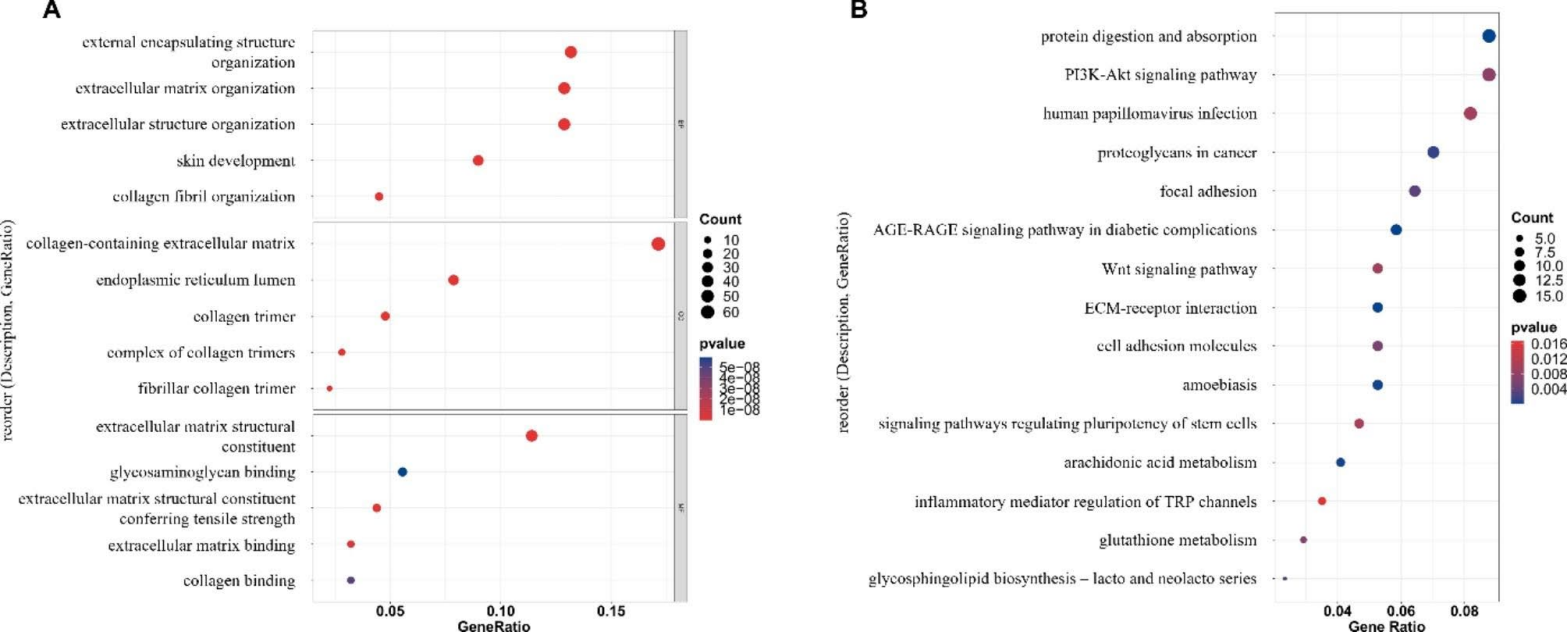
Biofunctional enrichment analysis of differentially expressed genes (DEGs) between oral mucosa (OM) samples and odontogenic keratocyst (OKC) samples. (**A**) Gene Ontology (GO) enrichment analysis of the DEGs. (**B**) Kyoto Encyclopedia of Genes and Genomes (KEGG) pathway enrichment analysis of the DEGs.

The above 402 differential genes were used for enrichment analyses based on GO and KEGG pathways to find the main functions and related pathways. All GO and KEGG terms enriched were shown in **Supplementary Tables 2** and **Supplementary Table 3**. GO terms consist of three parts: biological process (BP), molecular functions (MF), and cellular components (CC). DEGs linked to BP were mainly enriched in external encapsulating structure organization, extracellular matrix organization, extracellular structure organization, skin development, and collagen fibril organization. DEGs linked to CC were significantly enriched in collagen-containing extracellular matrix, endoplasmic reticulum lumen, collagen trimer, complex of collagen trimers, and fibrillar collagen trimer. DEGs linked to MF mainly focused on extracellular matrix structural constituent, glycosaminoglycan binding, extracellular matrix structural constituent conferring tensile strength, extracellular matrix binding, and collagen binding (Fig. 4A). Top 15 pathways enriched by KEGG were also presented, including protein digestion and absorption, PI3K-Akt signaling pathway, human papillomavirus infection, proteoglycans in cancers, focal adhesion, etc. (Fig. 4B).

### PPI Network Construction

**Fig. 5:**
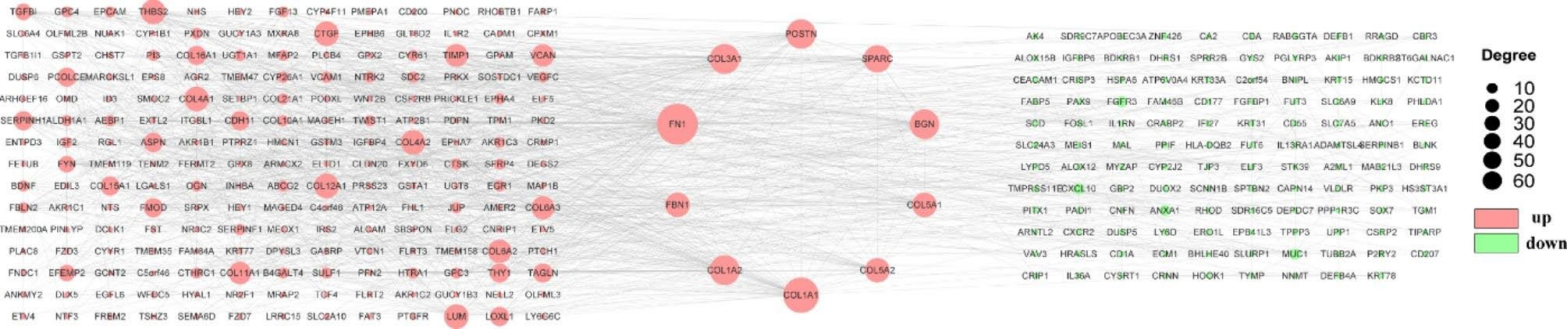
Interaction network and analysis of the hub genes. PPI network was constructed with the DEGs. The red points represent the upregulation of the expression of genes. The green points represent downregulated genes. The most significant module was obtained from PPI network with 10 nodes

The differential genes obtained were listed in the STRING database (https://cn.string-db.org/) to build the PPI network. Cytoscape was used to calculate the degree of each node (**Supplementary Table 4**). The top 10 genes were considered as the hub genes, including FN1, COL1A1, COL3A1, COL1A2, BGN, POSTN, SPARC, FBN1, COL5A1, and COL5A2 (Fig. 5). Given the fact that collagen-associated genes showed the predominance in the network and the enrichment results above as well, COL1A1 and COL3A1 were chosen for subsequent analyses.

### Immune Cell Infiltration Analysis

**Fig. 6:**
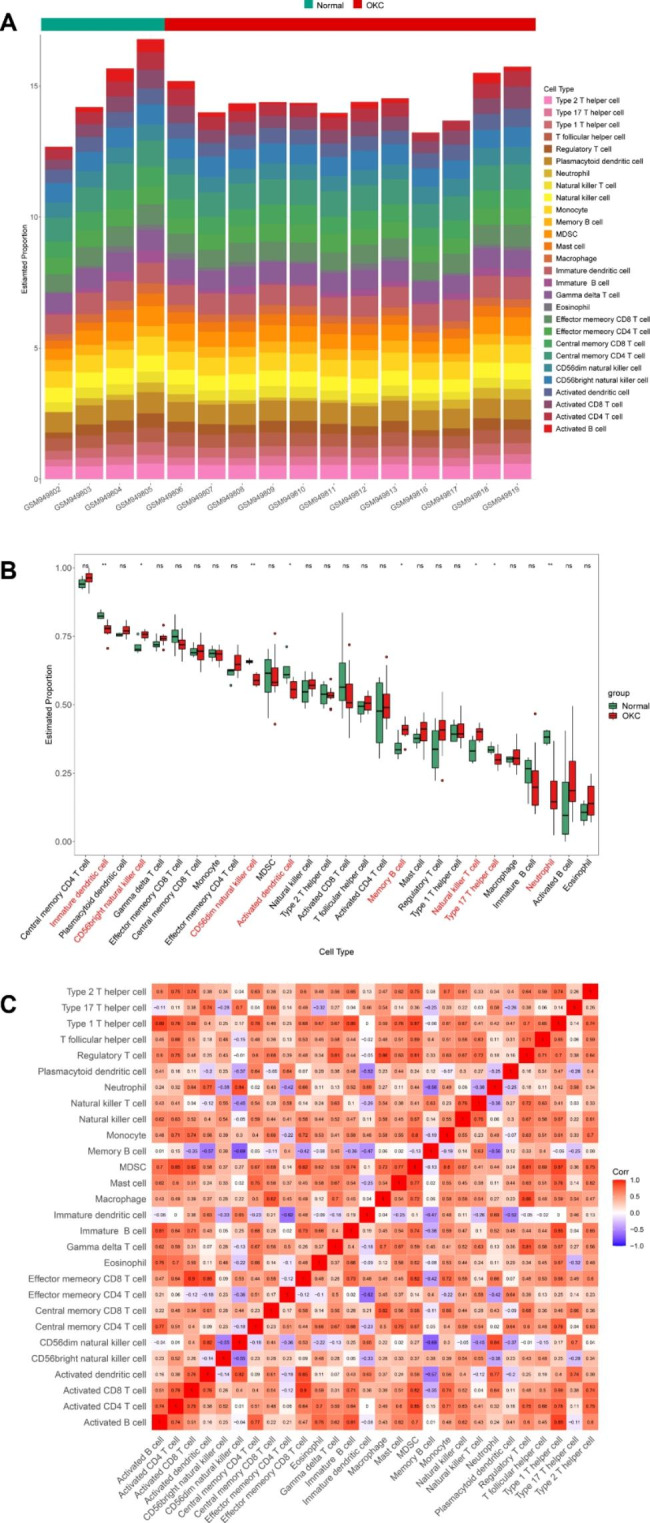
Immune cell infiltration patterns in oral mucosa (OM) samples and odontogenic keratocyst (OKC) samples. (**A**) Relative distribution of 28 immune cells in all samples. (**B**) Boxplot showing the differentially infiltrated immune cell types between OM group (green) and OKC group (red). ns, not significant, *P < 0.05, **P < 0.01. (**C**) Correlation heatmap of immune cell types in all samples. Red squares indicate positive correlation and blue squares indicate negative correlation, with deeper colored squares indicating stronger correlations

Notably, function enrichment analyses and PPI network construction identified multiple pathways and molecules related to ECM components organization. And as previously mentioned, collagen can be served as mediators for the regulation of various immune cell types, such as T cells and macrophages. Therefore, immune cell infiltration analysis was conducted between OM group and OKC group. The estimated proportions of 28 different immune cell types in each sample were calculated and shown in Fig. 6A. Of 28 immune cell types, 8 infiltrating cell types showed significantly different abundances between OM and OKC groups (Fig. 6B). Immature dendritic cells, CD56dim natural killer cells, activated dendritic cells, type 17 T helper cells, and neutrophils were found to show lower abundance in OKC samples. On the contrary, CD56bright natural killer cells, memory B cells, and natural killer T cells showed the opposite trend.

Furthermore, correlation analyses of infiltrated immune cells were conducted, with scores representing the degree of correlation (Fig. 6C). The correlation heatmap indicated that effector memory CD8 T cells and activated CD8 T cells showed the most synergistic effect, followed by that of type 1 T helper cell and activated B cell. On the other hand, memory B cells and CD56dim natural killer cells were mostly correlated in a negatively regulated manner.

### Analysis between hub genes and Immune Infiltration cells

**Fig. 7:**
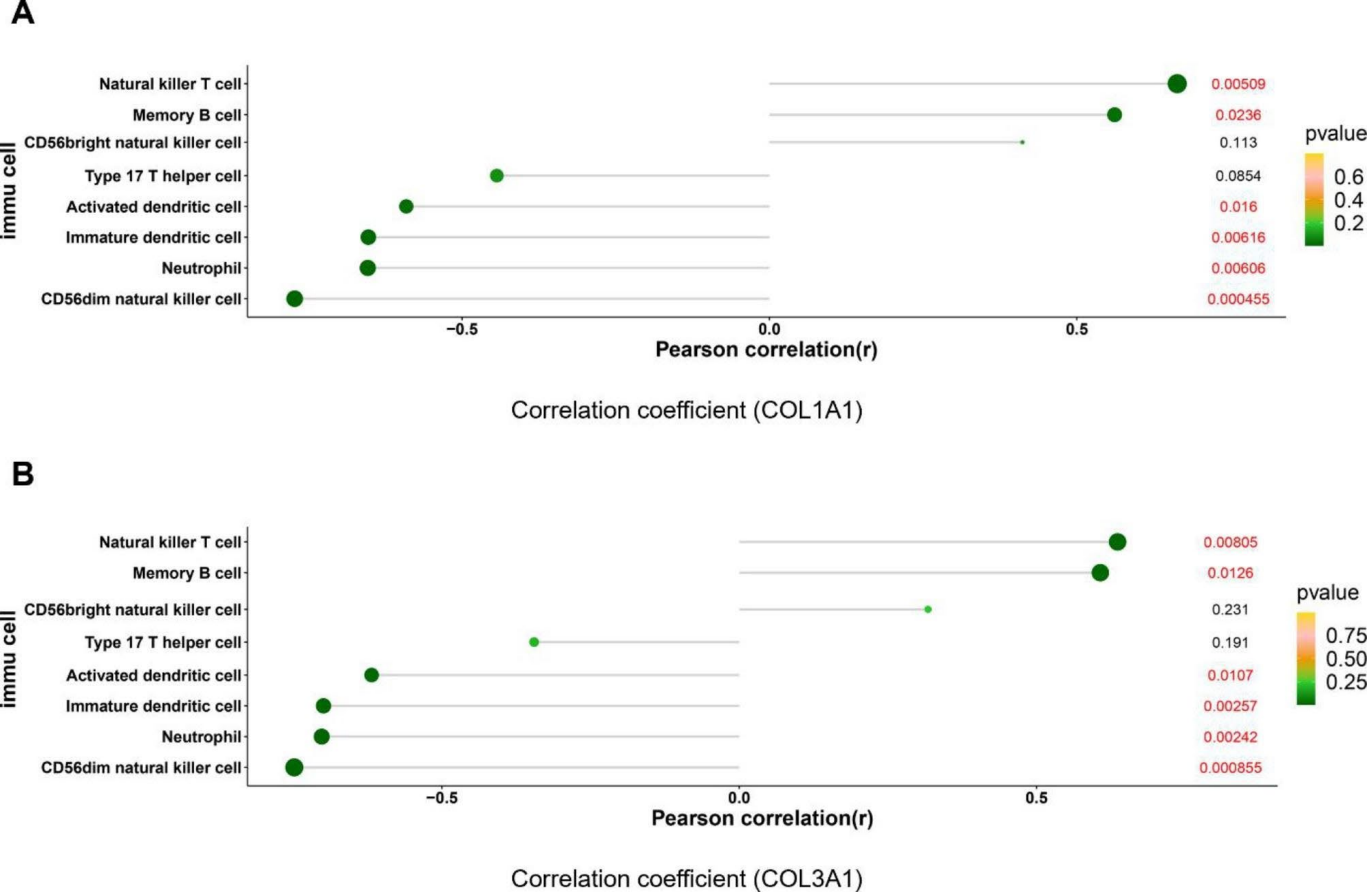
The Pearson’s correlation between hub genes COL1A1 (**A**) and COL3A1 (**B**) obtained above and the 8 different immune cell types was calculated. The size of the dots represents the strength of the correlation between genes and immune cells, and the color of the dots represents the *P*-value

The Pearson’s correlation analysis between the top 10 hub genes obtained above and the 8 immune cell types showing significantly different abundances was performed (Fig. 7**and Supplementary Fig. 1**). COL1A1 displayed a significant positive correlation with natural killer T cells (r = 0.663, *P* = 0.005) and memory B cells (r = 0.561, *P* = 0.0236), and a significant negative correlation with CD56dim natural killer cells (r = -0.772, *P* < 0.001), neutrophils (r = -0.653, *P* < 0.006), immature dendritic cells (r = -0.652, *P* = 0.006), and activated dendritic cells (r = -0.591, *P* = 0.016) (Fig. 7A). COL3A1 was positively corelated with natural killer T cells (r = 0.636, *P* = 0.008), memory B cells (r = 0.607, *P* = 0.013), and negatively correlated with CD56dim natural killer cells (r = -0.748, *P* < 0.001), neutrophils (r = -0.702, *P* = 0.002), immature dendritic cells (r = -0.699, *P* = 0.003), and activated dendritic cells (r = -0.618, *P* = 0.011) (Fig. 7B).

### Validation of COL1A1 and COL3A1 expression

**Fig. 8:**
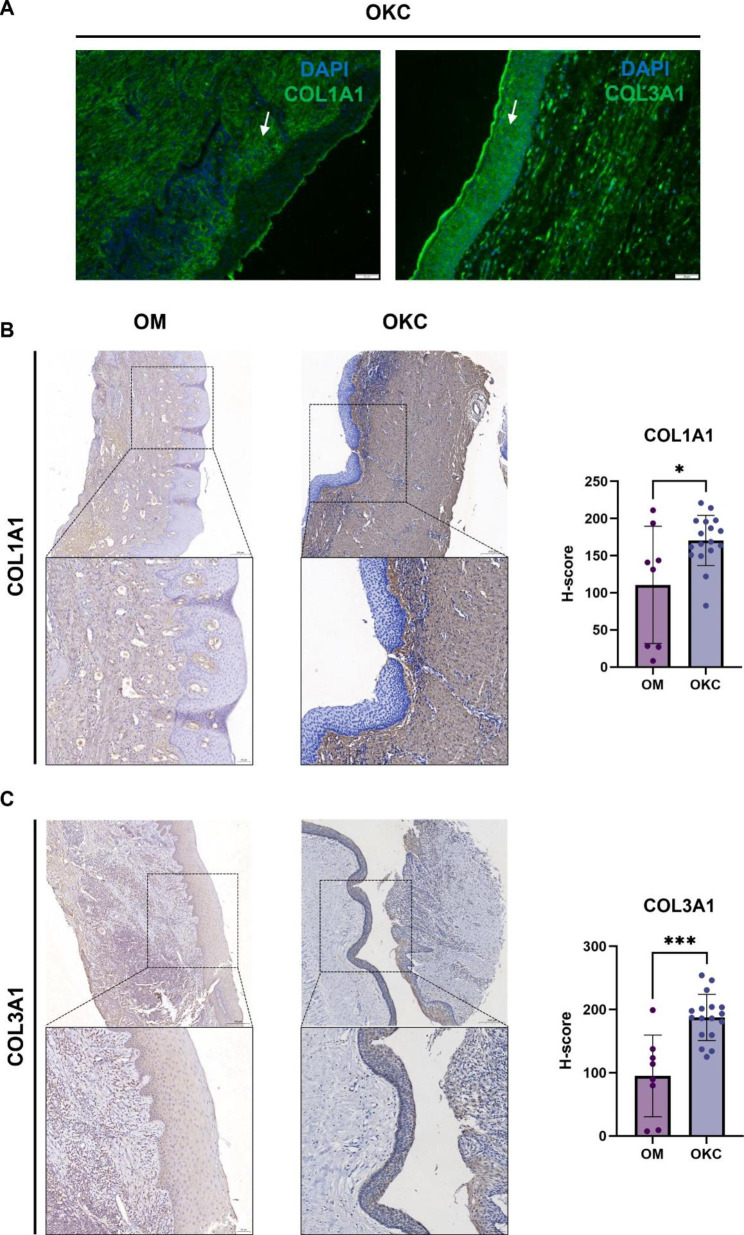
Validation of the hub genes in odontogenic keratocyst (OKC) samples and oral mucosa (OM) tissues. (**A**) Immunofluorescence assay showed the expression of COL1A1 and COL3A1 in OKC tissue. (**B**) Immunohistochemistry assay detected the expression of COL1A1 in OM and OKC tissues, and the differential analysis of COL1A1 in OM and OKC tissues. **P < 0.01. (**C**) Immunohistochemistry assay detected the expression of COL3A1 in OM and OKC tissues, and differential analysis of COL3A1 in OM and OKC tissues. *P < 0.05

IHC staining and IF staining were conducted to assess the expression and tissue distribution of COL1A1 and COL3A1. COL1A1 showed an obvious higher distribution in the stroma, while COL3A1 was mainly expressed in the epithelium visualized by IF staining (Fig. 8A). Of note, COL1A1 and COL3A1 expressions were both significantly higher in OKC samples than in OM samples (Fig. 8B-C).

## Discussion

Growing studies have focused on the high recurrence of OKC [3, 15]. Evidence has demonstrated that the expansion of various odontogenic cysts involves degradation of bone-matrix and cell attachment to extracellular matrix components [16]. It was reported that the collagen fibers in OKC were similar to those in odontogenic neoplasms, which proved the neoplastic behavior of OKC to some extent [17]. Moreover, a higher density of greenish-yellow collagen fibers was found in multilocular OKC than unilocular OKC, suggesting a strong aggressiveness of the former lesion [18].

Increasing evidence showed that the development of OKC was associated with immune cell infiltration [4]. In fact, immune cell infiltration is not only associated with inflammatory jaw cystic lesions such as radicular cyst, but also involved in the progression of OKC. Immune cell infiltration can be promoted by a variety of factors, such as inflammation, mechanical stress, chemokines, and cytokines. Alteration in the ECM can impact the recruitment of some immune cells, resulting in changes in the immune microenvironment [19].

PPI analysis screened hub genes that were broadly associated with other genes. The vast majority of these crucial genes were related to collagen, such as COL1A1, COL1A2, COL3A1, COL5A1, COL5A2, with the rest (FBN1, POSTN, SPARC, BGN) being components of the ECM. Theses hub genes may suggest that ECM components play a critical role in the development and progression of OKC. Although collagen has been demonstrated to affect T cells in tumor microenvironment [20], the role of COL1A1 and COL3A1 in promoting the migration and activity of immune cells has yet to be explored.

GO enrichment analysis showed that these differentially expressed genes were significantly enriched in the collagen-related pathways, such as collagen-containing extracellular matrix, collagen trimer, complex of collagen trimers, fibrillar collagen trimer, and extracellular matrix structural constituent, which showed an obvious difference in ECM components between OKC and OM. Previous studies have suggested that the differential distribution of the ECM proteins - fibronectin, tenascin, laminin, and collagen IV - in the cyst wall is responsible for the aggressive behavior of syndromic OKC [21], and fibroblasts in the cyst wall may also modulate the aggressive behavior by regulating ECM properties or epithelial-mesenchymal interactions [22]. These findings suggested that ECM components played critical roles in the aggressive behavior of OKC.

The above analyses showed the close relationships between the hub gene expression and immune cell infiltration. To further investigate the effect of immune cell infiltration in OKC, ssGSEA was used to perform a comprehensive analysis on OKC immune microenvironment. The results showed increased infiltration of CD56bright natural killer cell, memory B cell, and natural killer T cell, which may contribute to the occurrence and development of OKC.

Natural killer (NK) cells are an important component of innate immunity [23]. Phenotypically, two subsets of NK cells circulate in humans: CD56bright and CD56dim NK cells. CD56bright NK cells, also known as “helper” NK cells, are mainly responsible for the production of cytokines. While CD56dim NK cells are largely found in peripheral blood and directly kill target cells [24]. In the present study, CD56bright NK cell showed a higher abundance in OKC than that in OM, which may account for the high abundance of various kinds of cytokines [25]. The insufficient blood supply of odontogenic keratocyst tissues may be a factor in the lack of CD56dim NK cells.

Neutrophils, armed with a variety of “weapons” against pathogens, are a major branch of the innate immune system and are involved in almost all processes, such as acute injury and repair, cancer, autoimmunity, and chronic inflammatory processes [26]. The long-existing presence of microbial and biofilm-derived chemotactic and pro-inflammatory factors attract neutrophils from the circulation into the tissues, where they become activated [27], which could explain the infiltration of neutrophils in oral cavity. Besides, oral cavity is under continuous exposure of various mechanical stress from chewing, which may contribute to the recruitment of immune cells. However, in OKC lesions, a lower abundance of neutrophils was found possibly due to the closed cyst environment. Of note, although there are fewer neutrophils in OKC tissues than OM, it is reasonable to speculate that neutrophil infiltration might be higher in OKC tissues with infections and in OKC patients who have undergone the decompression surgery.

In the present study, the relationship between the immune cell types in OKC was investigated. The results showed that two pairs of immune cells (effector memory CD8 T cells & activated CD8 T cells, type 1 T helper cell & activated B cell) had the synergistic effect and that memory B cell and CD56dim natural killer cell were mostly correlated in a negatively regulated manner. However, the correlations between the immune cell types require further validation.

Collagen Type I Alpha 1 Chain (COL1A1) is the major component of type I collagen [28]. There were evidence of the involvement of members of the collagen family in carcinogenesis in several tissue types [29], and type I collagen are distributed around bone cells (osteoblasts, osteoclasts, osteocytes, etc.) as the most abundant organic component of the bone matrix [30]. Prostate cancer bone metastasis was previously shown to induce the destruction of collagen alignment in long bones [31]. Research suggested that the expression of COL1A1 was significantly associated with the infiltration level of immune cells, such as CD4 + T cells, CD8 + T cells, dendritic cells and neutrophils in low grade gliomas (LGG) [32]. In this study, the Pearson’s correlation analysis showed that COL1A1 displayed a significant positive correlation with NK T cell, central memory CD4 T cell, and effector memory CD4 T cell, which was consistent with the result in previous study, that was the positive correlation of T cell and COL1A1 [32]. On the other hand, the significantly negative correlations of COL1A1 with neutrophil and immature dendritic cell were observed, contradicting the previous conclusion [32]. In our study, it has been shown that COL1A1 was mainly distributed in stroma region and showed a significantly higher expression in OKC than in OM.

Collagen Type III Alpha 1 Chain (COL3A1) was found to be closely related to type I collagen [33]. In glioma, knockdown of COL3A1 could significantly inhibit the migration, invasion, and epithelial-mesenchymal transition (EMT) processes of tumor cells in vitro [34]. Immune cells can intimately interact with stromal cells and further impact tumor progression, invasion, and metastasis. A study showed that patients with head and neck squamous cell carcinoma (HNSCC) with increased COL3A1 levels also had a higher level of resting CD4 + T cells and resting NK cells [35]. The expression of COL3A1 was associated with the increase of IL-1β in osteoarthritis and was also linked to immune cell infiltration [36]. Interleukin- (IL-) 1β, one of the most powerful proinflammatory cytokines, is a strong stimulator of in vitro and in vivo bone resorption via upregulation of RANKL that stimulates the osteoclastogenesis [37]. Thus, it is conceivable that COL3A1 may be involved in the bone absorption and the progression of OKC through the elevation of IL-1β as well as immune cell infiltration. In our study, COL3A1 showed the strongest positive correlation with effector memory CD4 T cell, which was similar to a previous study [38]. In our IHC assay, it was also validated that the COL3A1 expression in OKC was significantly higher than in OM.

The aforementioned discussion highlights the significance of collagen genes, including COL1A1 and COL3A1, in the formation and remodeling of the extracellular matrix. The extracellular matrix, particularly in the context of tumors, has been linked with immune cell infiltration [19]. This matrix has the capacity to modify the behavior of immune cells, as well as regulate their recruitment and positioning within tissues. Consequently, changes in collagen production could potentially influence the infiltration and functionality of immune cells in OKC. We remain cautious about implying direct causation without further targeted studies, but these observations open up new avenues for future research.

While this study provides important insights into the role of collagen and immune cell infiltration in the development and progression of OKC, it has several limitations. First, our findings are primarily based on statistical analysis of existing datasets and require further experimental validation and expanded sequencing datasets valiadation. The expression levels and role of the identified hub genes in influencing the immune cell migration and activity need to be explored with additional in vitro and in vivo studies. Second, the examination of immune cell infiltration was performed using ssGSEA, which provides an estimation rather than a direct measurement of immune cell abundance. Future studies employing techniques such as flow cytometry or immunohistochemistry could provide a more accurate picture of immune cell infiltration in OKC. Lastly, this study largely focuses on collagen-associated genes and pathways. However, other ECM components and molecules involved in cell-matrix interactions might also play significant roles in OKC’s development and progression. These elements were not thoroughly addressed in our study and should be investigated in future research. These limitations notwithstanding, our findings shed light on the potential role of the ECM and immune cell infiltration in the aggressive behavior of OKC and provide a foundation for future investigations.

## Conclusions

Through bioinformatics, this study identified key hub genes and pathways integral to OKC development and distinct immune cell infiltration differences between OKC and OM samples. The genes COL1A1 and COL3A1, validated via IHC and IF assay, showed high expression in OKC tissues and correlated with immune cell infiltration. These findings elucidate OKC pathogenesis mechanisms, suggesting COL1A1 and COL3A1 as potential immune-related prognostic markers, pending further validation with larger samples.

## Electronic supplementary material

Below is the link to the electronic supplementary material.

Supplementary Material 1

Supplementary Material 2

Supplementary Material 3

Supplementary Material 4

Supplementary Material 5

## Data Availability

The dataset (accession number: GSE38494) analyzed in this study can be found in the Gene Expression Omnibus (https://www.ncbi.nlm.nih.gov/geo).

## Abbreviations

BP: biological process
CC: cellular components
COL1A1: Collagen Type I Alpha 1 Chain
COL3A1: Collagen Type III Alpha 1 Chain
DCs: dendritic cells
DEGs: differentially expressed genes
ECM: extracellular matrix
EMT: epithelial-mesenchymal transition
FC: fold change
GEO: Gene Expression Omnibus
GO: Gene Ontology
H-score: histo-score
HNSCC: head and neck squamous cell carcinoma
IF: immunofluorescence
IHC: immunohistochemistry
IL-: interleukin-
KEGG: Kyoto Encyclopedia of Genes and Genomes
LGG: low grade gliomas
MF: molecular functions
NK: natural killer
OKC: odontogenic keratocyst
OM: oral mucosa
PCA: principal component analysis
PPI: protein-protein interaction
ssGSEA: single sample gene set enrichment analysis
STRING: Search Tool for the Retrieval of Interacting Genes
