# Supplementary material for: Identification of Immune Infiltration in Odontogenic Keratocyst by Integrated Bioinformatics Analysis
Source: BMC Oral Health. 2023 Jul 6;23:454. doi: 10.1186/s12903-023-03175-9 (PMC10324234; doi:10.1186/s12903-023-03175-9)
Supplement: Supplementary file 5 — Supplementary Material 5 [file 12903_2023_3175_MOESM5_ESM.docx]

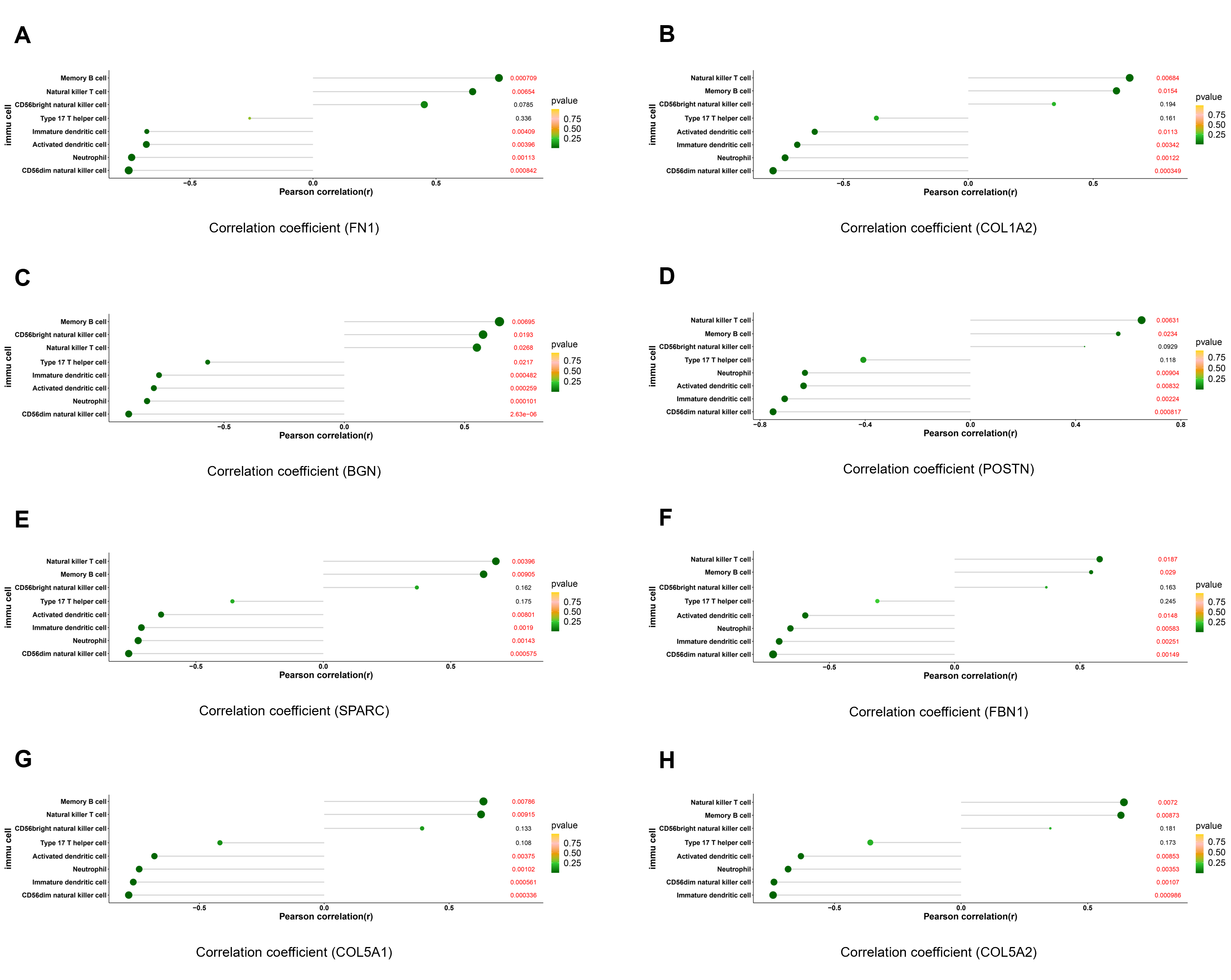


**Supplementary Figure 1. Correlation between 8 hub genes and 8 infiltrating immune cell types.** The size of the dots represents the strength of the correlation between genes and immune cells, and the color of the dots represents the P-value.
